# Comparative analysis of sequencing technologies for single-cell transcriptomics

**DOI:** 10.1186/s13059-019-1676-5

**Published:** 2019-04-09

**Authors:** Kedar Nath Natarajan, Zhichao Miao, Miaomiao Jiang, Xiaoyun Huang, Hongpo Zhou, Jiarui Xie, Chunqing Wang, Shishang Qin, Zhikun Zhao, Liang Wu, Naibo Yang, Bo Li, Yong Hou, Shiping Liu, Sarah A. Teichmann

**Affiliations:** 1 Wellcome Sanger Institute, Wellcome Genome Campus, Hinxton, Cambridge, CB10 1SA UK; 2 European Bioinformatics Institute (EMBL-EBI), Wellcome Genome Campus, Hinxton, Cambridge, CB10 1SD UK; 3 Danish Institute of Advanced Study (D-IAS), Functional Genomics and Metabolism Unit, Department of Biochemistry and Molecular Biology, University of Southern Denmark, Odense, 5230 Denmark; 4 BGI-Shenzhen, Shenzhen, 518083 China; 5 State Key Laboratory of Bioelectronics, Southeast University, Nanjing, 210096 China; 6 China National GeneBank, BGI-Shenzhen, Shenzhen, 518120 China; 7 School of Biology and Biological Engineering, South China University of Technology, Guangzhou, China; 8 Theory of Condensed Matter, Cavendish Laboratory, Cambridge University, JJ Thomson Avenue, Cambridge, CB3 0HE UK

**Keywords:** Single-cell RNA sequencing, Sequencing platforms, Benchmarking scRNA-seq, Illumina sequencing, BGISEQ-500

## Abstract

Single-cell RNA-seq technologies require library preparation prior to sequencing. Here, we present the first report to compare the cheaper BGISEQ-500 platform to the Illumina HiSeq platform for scRNA-seq. We generate a resource of 468 single cells and 1297 matched single cDNA samples, performing SMARTer and Smart-seq2 protocols on two cell lines with RNA spike-ins. We sequence these libraries on both platforms using single- and paired-end reads. The platforms have comparable sensitivity and accuracy in terms of quantification of gene expression, and low technical variability. Our study provides a standardized scRNA-seq resource to benchmark new scRNA-seq library preparation protocols and sequencing platforms.

**Electronic supplementary material:**

The online version of this article (10.1186/s13059-019-1676-5) contains supplementary material, which is available to authorized users.

## Background

Single-cell RNA-seq (scRNA-seq) has become the established approach to dissect cellular heterogeneity, unravel cell states, and identify subpopulation structures across different cell types [1–4]. The different scRNA-seq methods and technologies have been benchmarked using synthetic RNA spike-ins [5–7]. However, to date, most scRNA-seq methods require cDNA libraries to be compatible with short-read Illumina sequencing platform.

The most widely used Illumina platform uses a stepwise sequencing by polymerase approach. The libraries are made by fragmentation of bulk or single-cell cDNA, followed by the addition of custom adaptors. The template is flooded across a patterned flow cell to bind with immobilized primers and washed to remove unbound ends. The bound but free template ends further interact with nearby primers, forming bridge structures. The second strand is synthesized by PCR using the same primers, followed by washing and re-formation of bridges. This bridge amplification typically generates more than a million copies of each template within the tight physical cluster on the flow cell (reviewed in [8]). A simplified schematic is shown in Additional file 1: Figure S1A. The actual sequencing process itself is termed “sequencing by synthesis.” Here, a mixture of primers, DNA polymerase, and modified nucleotides are added to enriched template on flow cell. During each cycle, fragments within each cluster incorporate a complementary single modified nucleotide with a base-specific, cleavable fluorophore, while unbound fragments are washed away. The flow cell is imaged using total internal reflection fluorescence (TIRF) microscopy to identify incorporated based, followed by cleavage of modified base. This cycle of nucleotide addition, elongation, and cleavage is repeatedly performed to ascertain the DNA sequence.

The BGISEQ-500 is an alternative short-read sequencing platform, developed by BGI (Beijing Genomics Institute). The BGISEQ-500 works use combinatorial probe-anchor synthesis (cPAS) that combines DNA-Nanoball (DNBs) arrays with stepwise sequencing using DNA polymerase on a flow cell [8] (Additional file 1: Figure S1A). The three key steps in the BGISEQ-500 platform are generation of DNBs, loading DNBs onto a flow cell, and the sequencing of DNA fragments. In cPAS-based sequencing, the template cDNA is first fragmented and size selected (200–500 bp). The template undergoes four sequential rounds of adaptor ligation, circularization, and cleavage, generating a final circularized template with four unique adaptors. The circular templates undergo rolling circle amplification (RCA) to produce a large mass of DNA concatemers (DNBs) and are finally immobilized and sequenced on a flow cell using combinatorial probe-anchor synthesis (cPAS). Across the flow cell, the DNBs bind to an anchor and fluorescent probe (complementary to adaptors). The probes are degenerate (apart from the first position) and capture the first base at either end of the anchor. Each sequencing cycle consists of removing the previous probe, re-ligating to the same anchor with different fluorescent probes, and sequence determination. This cycle is repeated for each of the remaining three adapter sequences to generate paired-end reads (reviewed in [8]). The BGISEQ-500 platform has been previously applied to detection of small noncoding RNAs [9], human genome re-sequencing [10], and palaeogenomic ancient DNA sequencing [11], but not to scRNA-seq.

One of the key differences between the BGISEQ-500 and Illumina platforms is the sequencing cost, calculated from yield per run. These sequencing costs are typically subject to geographical, institutional pricing and continue to decline. Typically, the cost per gigabase (Gb) on BGISEQ-500 is 40–60% of Illumina HiSeq4000 platform, without accounting for physical sequencer cost (Additional file 3: Table S1). The combination of higher throughput (~ 2×) with marginally increased cost (10–15%) per lane makes the BGISEQ-500 an attractive alternative. This is important in scRNA-seq, where significant multiplexing is required alongside considerable read depth per cell.

Here, we assess the suitability of BGISEQ-500 sequencing platform for scRNA-seq and compare with the Illumina HiSeq platform using matched single-cell data.

We perform two different scRNA-seq methods (SMARTer and Smart-seq2) on mouse embryonic stem cells (mESCs) and human K562 cells [12, 13]. We chose mESCs and K562 cells as two widely used mouse and human cell lines, which have been profiled in large-scale consortia (e.g., ENCODE) and in studies benchmarking single-cell protocols [5, 6, 14]. For comparison, we utilize RNA-spike-ins including External RNA Controls Consortium (ERCCs) and Spike-in RNA Variants (SIRVs). The ERCCs and SIRVs span 92 synthetic RNA species and 69 artificial transcripts, respectively, of varying lengths, concentrations, GC contents, isoforms, and abundance levels. We benchmark and compare two performance metrics (*sensitivity* and *accuracy*) on single cells using two different protocols and across Illumina and BGISEQ-500 sequencing platform.

We have previously applied these performance metrics to compare different scRNA-seq protocols [5]. As in our previous framework, the “sensitivity” or molecular detection limit is defined as the minimum number of RNA spike-in molecules detected within a single cell. The “accuracy” refers to the correlation between the estimated abundances of input RNA spike-ins and the known input molecules added to single-cell reaction (ground truth) (Additional file 1: Figure S1B–C). Specifically, the single-cell sensitivity is computed using a logistic regression model with spike-in RNA detection as a dependent variable across platforms. The sensitivity is measured as the input spike-in abundance level, where the detection probability reaches 50% (Additional file 1: Figure S1B). This approach minimizes biases due to batch effects (uneven sizes, sampling, and variable spike-in detection). The accuracy is calculated using the Pearson product-moment correlation coefficient (*R*) between estimated spike-in expression from sequencing and the a priori known input spike-in concentration (ground truth) in log space, for each individual cell (Additional file 1: Figure S1C, Additional file 2: Supplementary methods).

In this study, we perform the first systematic scRNA-seq comparison across two sequencing platforms, using 1297 matched cDNA samples from 468 unique single cells using two scRNA-seq protocols. We compare and assess the accuracy, sensitivity, and robustness of BGISEQ-500 library preparation and sequencing platform with the current state-of-art Illumina HiSeq platform. Our large dataset contains single- and paired-end reads (50 and 100 bp) for batch-matched mESCs and K562s, which is a large data resource for comparison of new protocols and benchmarking computational methods.

## Results

We performed two scRNA-seq protocols (SMARTer and Smart-seq2) in parallel on 288 single-mESCs using both ERCCs and SIRVs spike-ins on Fluidigm C1-system [12, 13]. The Smart-seq2 protocol was performed in replicates (SM2 replicate 1 and 2) using mESCs batches. The single-cell lysis, reverse transcription, and pre-amplification for all methods were done within the C1-system. Each chamber within the C1 chip was visually validated to contain a single cell and to avoid bad chambers (doublet, debris, and dead cells; Additional file 2: Supplementary methods).

**Fig. 1:**
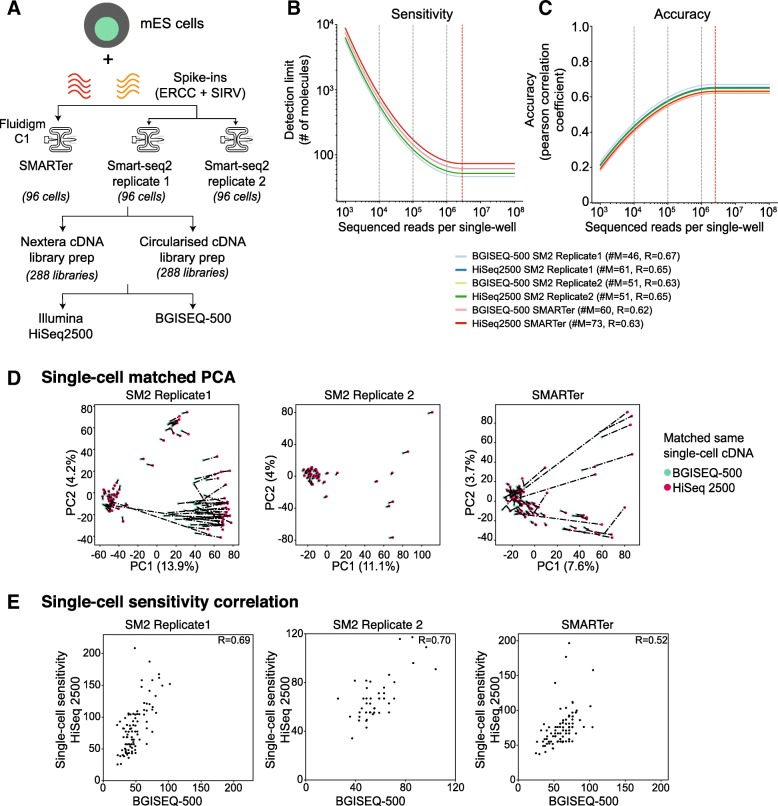
**a** Schematic overview of the mESC scRNA-seq experiment and sequencing. Three sets of 96 mESCs are profiled using SMARTer and Smart-seq2 protocols on C1-system. For each single-cell, we prepared two sets of libraries for Illumina and BGISEQ-500 platform resulting in 576 matched libraries. **b** Single-cell detection limit (Sensitivity) of mESC cells, downsampled across two orders of magnitude. The single-cell sensitivities are largely similar between different library preparations across scRNA-seq protocols. **c** Single-cell accuracy of mESC cells also downsampled across two orders of magnitude. The grey dotted lines in **b** and **c** indicate downsampled single cells at different depths, while red line indicates limit for sequencing saturation. **d** PCA for matched single-cells performed using SMARTer and two replicates of Smart-seq2 and sequenced across both sequencing platforms. Each single cell is represented with a red and green colored circle to indicate HiSeq200 and BGISEQ-500 sequencing platforms respectively. The dotted lines represent distance, i.e., measure of similarity across sequencing platforms. **e** Single-cell correlations of sensitivity (i.e., lower detection limit computed from spike-in concentrations) for each scRNA-seq protocol and across sequencing platforms. The correlations (*R* = 0.52~0.70) are comparable between sequencing platforms

We used the same “matched” single-cell cDNA from 288 cells, across SMARTer and Smart-seq2 protocols, and generated 576 single-cell libraries for both Illumina HiSeq2500 and BGISEQ-500 platforms (Fig. 1a) [5]. Here, we have both matched cDNA from single cells as well as single-cell replicates to study how different library preparation and sequencing platforms affect single-cell measurements (Fig. 1a). The final libraries had similar size distributions across both sequencing platforms, though there is a weak trend for slightly larger fragments in the BGISEQ-500 libraries.

For each single cell, we converted the aligned reads to normalized transcript per million (TPM) units (Additional file 2: Supplementary methods). Across single cells, the fragment size distribution, read coverage over genes, dropout rates, and expression variation were quite similar between both sequencing platforms (Additional file 1: Figure S1D). We also devised pseudo-bulk by pooling all single cells together and observed high correlations between sequencing platforms and protocols (Additional file 1: Figure S1E).

Next, we calculated the sensitivity and accuracy using both sets of spike-ins across matched single cells across both platforms (Additional file 1: Figure S2A–B). Globally, the single-cell accuracy was similar across the sequencing platforms, irrespective of scRNA-seq protocol (*R* = 0.66–0.70, Additional file 1: Figure S2B; violin plots in Additional file 1: Figure S2C). The sensitivity (i.e., detection limit) was similar across scRNA-seq protocols and ranged from 21 to 47 molecules (number of RNA molecules *#M* = 21–47, Additional file 1: Figure S2A; violin plots in Additional file 1: Figure S2D) between sequencing platforms. The detection limit was slightly lower for BGISEQ-500 platforms, likely due to highly sequencing depth. Surprisingly, we could detect as few as 12 molecules across one of the Smart-seq2 protocol replicates (SM2-seq replicate 1; Additional file 1: Figure S2D).

Given that sensitivity can be dependent on sequencing depth [5], we compared the distribution of reads across single cells. The single cells were more deeply sequenced across BGISEQ-500 platform, accounting for slightly increased sensitivity (Additional file 1: Figure S2E). This also increased the detected genes in BGISEQ-500 compared to Illumina platform (Additional file 1: Figure S2F). Interestingly, we cultured these mESCs in media containing serum and LIF (leukemia inhibitory factor), where stem cell and differentiating subpopulations have been identified [15, 16]. In one of our Smart-seq2 protocol replicates (SM2-seq replicate1), we could discern two subpopulations based on the number of genes (Additional file 1: Figure S2F). We classified all single cells based on the number of genes expressed across platforms (Illumina > 5000 and BGI > 7500 genes) and observed that these subpopulations expressed different levels of pluripotency markers. The more pluripotent cells (Illumina > 5000 and BGI > 7500 genes) expressed higher levels of stem cell markers (Illumina > 5000 and BGI > 7500 genes), while the differentiated-like cells had fewer expressed genes and lower, stochastic gene expression (Additional file 1: Figure S2F–G) [15, 16]. The statistics (performance metrics, reads, spike-in, genes detected, etc.) for each matched single cell are summarized in Additional file 4: Table S2.

To reduce the bias including technical variability, due to sequencing depth, we downsampled total reads across two orders of magnitude (raw reads to 10^6^, 10^5^, 10^4^ total reads) and re-computed sensitivity and accuracy. This allows us to compare the platforms at different sequencing depths and also estimate where saturation of sequencing occurs. Both sensitivity and accuracy were highly similar across single cells between scRNA-seq protocols and sequencing platforms, upon downsampling (Fig. 1b–c). The detection limit was consistent between scRNA-seq protocols and sequencing platforms (*#M* = 46–73), with the sensitivity reaching saturation around ~ 2.8 million reads (red dashed line; Fig. 1b). The accuracy was also consistent (*R* = 0.62~0.67) between platforms, reaching saturation at ~ 2.5 million reads (red dashed line; Fig. 1c).

We next assessed the matched single-cell similarity across platforms by comparing either total expression (Genes + spike-ins) or for spike-ins alone. We accounted for sequencing depth by downsampling to 1 million reads per single cell, as this has shown to be enough for single-cell analysis [5]. We performed principal component analysis (PCA) and plot each matched cell by representing the sequencing platforms, where the distance between them is a measure of gene expression similarity (dashed line; Fig. 1d). The PCA captures strong similarity between matched cells (short distance) across platforms for most of the single cells across both platforms, with low PC1 (8–14%) and PC2 (4%) contribution. The PCA separates the pluripotent subpopulation from outlier cells (bad cells) (Fig. 1d). This is most apparent in Smart-seq2 protocol replicate 1 (Fig. 1d; first panel), where the largest subpopulation (left) corresponds to pluripotent cells, while the smallest subpopulation is differentiating-like cells (top-center; see also Additional file 1: Figure S2G) with a group of low-quality outliers (with large inter-cell distances; see also Additional file 1: Figure S2G).

We re-performed PCA using both sets of spike-ins (without genes) on matched single cells to re-validate the subpopulations and to control for any technical bias arising from endogenous genes. As expected, the matched cells in resulting PCA were uniform with similar distances and low PC1 (~ 7%) and PC2 (~ 5–6%) variation between platforms (Additional file 1: Figure S3A). Across Smart-seq2 protocol replicate 1, we could re-confirm the two subpopulations (same as in Additional file 1: Figure S2F–G). The larger subpopulation corresponds to pluripotent cells expressing more genes with fewer spike-ins detected, while the other subpopulation had higher spike-ins. We also compared the single-cell sensitivities for matched cells both before and after downsampling. The rationale is that without downsampling, single-cell correlations would be poorer due to sequencing depth variation, and skewed towards the more deeply sequenced BGISEQ-500 data. As expected, the correlations were poorer before downsampling (*R* = 0.14~0.52; Additional file 1: Figure S3C). Upon downsampling, the correlations were significantly improved in a manner that is consistent across protocols (*R* = 0.52~0.70).

We also compared the single-cell sensitivities for matched cells both before and after downsampling. The rationale is that without downsampling, single-cell correlations would be poorer due to sequencing depth variation and skewed towards the more deeply sequenced BGISEQ-500. As expected, the correlations were poorer before downsampling (*R* = 0.14~0.52, Additional file 1: Figure S3C). Upon downsampling, the correlations were significantly improved in a manner that is consistent across protocols (Fig. 1e, *R* = 0.52~0.70).

In summary, we highlight that both Illumina and BGISEQ-500 platforms have similar and comparable performance metrics (sensitivity and accuracy) and can capture underlying biological subpopulations at single-cell level. The BGISEQ-500 offers a cost-effective alternative to Illumina platform with similar yields.

**Fig. 2:**
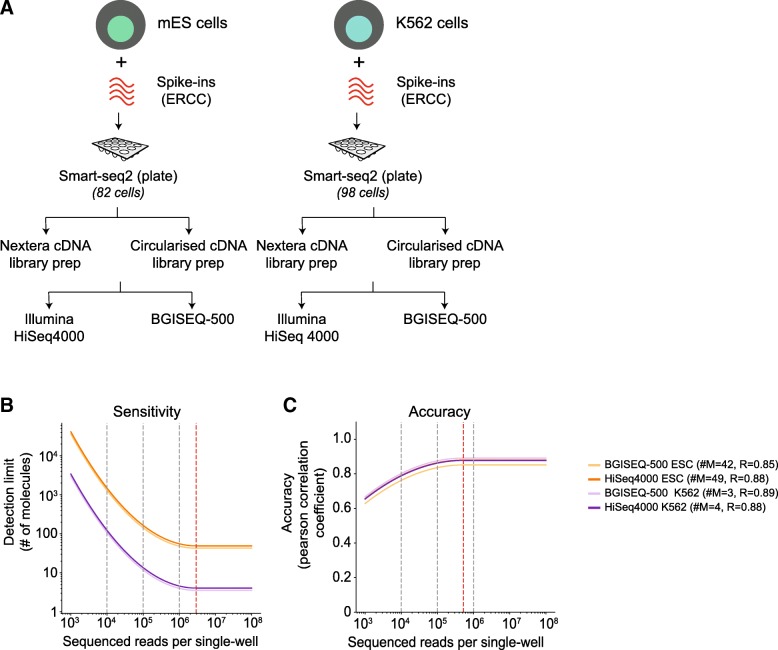
**a** Schematic overview of the mESC and K562 scRNA-seq experiment using plate-based Smart-seq2 protocol and sequencing. 82 mESCs and 98 K562s are profiled using plate-based Smart-seq2 protocols, followed by matched single- and paired-end library preparation for both Illumina and BGISEQ-500 platform resulting in 721 matched libraries. **b** The sensitivity of mESCs and K562s cells downsampled across two orders of magnitude. The sensitivity is critically dependent on sequencing depth and deeply sequenced K562 cells appear to be more sensitive than mESCs. The sensitivity, however, between the sequencing platforms is highly similar. **c** The accuracy of mESCs and K562s downsampled across two orders of magnitude. The accuracy has little dependence on sequencing depth, and both mESCs and K562 have similar accuracies, across both sequencing platform. The grey dotted lines indicate downsampling at different read depths per cell, while red line indicates saturation per cell

Next, we repeated our benchmarking comparison using plate-based Smart-seq2 protocol on a smaller subset of 82 mESCs and 98 K562s using ERCC spike-ins only. We chose plate-based Smart-seq2 as its most widely used full-length protocol and avoid cell capture biases in the C1-platform. We used the matched single-cell cDNA from mESCs and K562s to generate 600 BGISEQ-500 sequencing libraries in both single- and paired-end configurations and 121 HiSeq 4000 paired-end sequencing libraries (721 sequencing libraries in total) (Fig. 2a; Additional files 4, 5, 6, and 7: Table S2, S3, S4, and S5). This setup allows us to compare the effect of library preparation and sequencing platform from both matched single-cell cDNA, but also single-cell replicates processed in parallel (Fig. 2a).

Across single-cells, the sequencing depth varied from 3 to 20 million reads across both sequencing platforms. The mESCs are much smaller than K562s with dramatically variable amount of cellular RNA per single cell. Across both sequencing platforms, we observed higher sequencing depth and higher number of genes and spike-ins detected for K562s compared to mESCs (Additional file 1: Figure S4A; Additional file 6: Table S4). Taking a conservative cutoff (TPM > 10), we observed most genes (~ 70%) were expressed in both cell types, as well as cell type-specific genes (5500 and 2371 genes across K562s and mESCs) (Additional file 1: Figure S4H).

Owing to the higher sequencing depth, the K562s had increased sensitivity across both sequencing platforms with detection of as few as 3–4 molecules (#M = 3–4; Fig. 2b). This compared to a somewhat worse but consistent mESC detection limit (#M = 42~49), due to sequencing depth variation. The accuracy, which is less dependent on depth was quite high and consistent (*R* = ~ 0.85~0.88) for both cell types, indicating similar performance metrics across sequencing platforms (Fig. 2b and Additional file 1: Figure S4B–C). The accuracy saturated at ~ 0.5 million reads per single cell. The statistics (performance metrics, reads, genes, spike-ins detected, etc.) for each matched K562 and mESCs across both platforms are provided in Additional files 4, 5, 6, 7, and 8: Table S2, S3, S4, S5, and S6.

Since we generated mESC data from different scRNA protocols, technologies, and sequencing platforms containing ERCC spike-ins in two different batches, we collectively assessed the mESCs performance metrics for both platforms. We downsampled the raw reads and observed that both sensitivity and accuracy were comparable between both platforms (Additional file 1: Figure S4D–E). Combining all the mESC data, the sensitivity and accuracy were saturated at ~ 2 million and ~ 250,000 reads, respectively (Additional file 1: Figure S4D–E). We also observed similar detected genes detected between both platforms (Additional file 1: Figure S4E, Additional files 4 and 5: Table S2 and S3). In summary, our analysis demonstrates similar and robust performance metrics between BGISEQ-500 and Illumina platforms for scRNA-seq.

Our dataset spans 468 unique single cells of two different cell types (mESCs, K562s), two scRNA-seq protocols (SMARTer, Smart-seq2), two technologies (Fluidigm C1, plate-based), and matched 1297 libraries across Illumina and BGISEQ-500 sequencing platform (Additional files 4, 5, 6, 7, and 8: Table S2, S3, S4, S5, and S6). In addition to paired-end (PE) data, we also generated 50 bp and 100 bp single-end (SE) scRNA-seq from mESCs and K562 totaling > 750 GB of raw single-cell data.

From both the SE and PE BGISEQ-500 data, the average sequencing depth per cell was ~ 9.6 and ~ 8.7 million reads, respectively (Additional files 5, 6, 7, and 8: Table S3, S4, S5 and S6). Both SE and PE datasets detected > 9500 genes for K562s and > 9000 genes for mESCs. The accuracies for K562 and mESCs cells across both SE and PE reads was *R* = ~ 0.70–0.85, and the sensitivities for K562 and mESCs cells were #M = 4~25 across both SE and PE reads. We also compared the frequency of alternative splicing events in the downsampled mESCs and K562s PE data. We observe quite similar alternative splicing events between K562 and mESCs across both platforms (Additional file 1: Figure S4G).

In summary, our datasets and comparative performance assessment offer a large standardized resource to the community to further investigate potential technical biases including GC content, isoform quantification, impact of read-lengths across different scRNA-seq protocols, technologies, and sequencing platforms. The matched 1297 single-cell datasets and annotations would serve as an ideal starting point for benchmarking and comparison of new protocols and computational methods for the scientific community.

## Discussion

The rapid developments in single-cell genomics are transforming our understanding of biological systems by capturing underlying gene expression variability to identify cell types, states, and transitions across cell populations. Single-cell transcriptomic profiling is a multi-step sampling procedure, where the first major step involves cell lysis, RNA capture, reverse transcription of RNA, preamplification of cDNA generation. The next major step requires single-cell cDNA to be converted into a sequencing compatible library, followed by sequencing. There are several scRNA-seq protocols that utilize different chemistries, platforms, and technologies to address the first critical step of converting RNA into cDNA. The technical variation, performance metrics (sensitivity, accuracy), and reproducibility for the first critical step have been recently evaluated and benchmarked using synthetic RNA spike-in molecules [5–7]. However, all the scRNA-seq protocols and technologies require libraries to be compatible for sequencing on short-read Illumina platform.

Here we explore an alternative BGISEQ-500 short-read sequencing platform for scRNA-seq that uses combinatorial probe-anchor synthesis (cPAS). Unlike Illumina, BGISEQ-500 platform performs template enrichment using rolling circle amplification on DNA-Nanoballs combined with stepwise sequencing for template amplification [8] (Additional file 1: Figure S1A). One of the biggest advantages of BGISEQ-500 is the cost and throughput per run (Gb/per run). It is important to highlight that the sequencing costs continue to decline yearly and reagent and personnel costs across facilities are often subject to geographical and institutional pricing, making it difficult to compare exact costs. However, a typical BGISEQ-500 100 bp paired-end run generates 120–130 Gb (1.8-2x Illumina throughput) at 10–15% increased cost per lane. This can be especially useful for full-length scRNA-seq, where both multiplexing and higher sequencing depth per cell is required. On the other hand, Illumina platform is the current state of the art with reagents widely available, used, and benchmarked.

Our study is the first to utilize BGISEQ-500 platform for scRNA-seq. Our comprehensive benchmarking of performance metrics utilizes two scRNA-seq protocols (SMARTer and Smart-seq2), multiple spike-ins (ERCC alone, ERCC+SIRV), two different cell lines (mESCs, K562s), and two technologies (Fluidigm C1, plate-based) across Illumina HiSeq and BGISEQ-500 platform. Utilizing 468 single K562 and mESCs and matched 1297 single-cell libraries, we observe BGISEQ-500 to be highly comparable in sensitivity, accuracy, and reproducibility to Illumina platform, while being considerably more cost-effective.

From our mESC scRNA-seq dataset, we could distinguish technical artifacts (sequencing depth) from biological variation (subpopulations) across both sequencing platforms. We observe differential alternative splicing events between K562s and mESCs across both sequencing platforms. We observe some RNA degradation in few single-cell libraries, which we believe is due to transport of samples. Our data using mESCs and K562s across two scRNA-seq protocols supports the notion that minimal variability is introduced during library preparation and sequencing for both Illumina and BGISEQ-500 platforms. In combination with our previous framework [5], we believe that variability between the steps of scRNA-seq protocols is largest during the RNA to cDNA step. Both the performance metrics and single-cell characteristics (number of genes, expression range, subpopulation, etc.) suggest that BGISEQ-500 library preparation and sequencing are robust and comparable to Illumina platforms for single-cell applications.

We observe minimal variability in cDNA processing across different library preparation and sequencing platforms. In the current study, we did not perform scRNA-seq protocols with Unique Molecular Identifiers (UMIs) that account for PCR amplification biases. Given that UMIs primarily address biases during the RNA-to-cDNA stage (and to cDNA amplification), this would have minimal or no impact on our assessment of sequencing platforms. The scRNA-seq UMI-based protocols could easily be extended to be compatible with sequencing on BGISEQ-500 platform. Our large resource for benchmarking scRNA-seq data suggests that the BGISEQ-500 platform is suitable for plate-based (microwell or nanowell), droplet, and microfluidics technologies.

In addition to benchmarking, we provide a large comprehensive multi-cell type, protocol, and platform scRNA-seq dataset spanning 468 cells and 1297 libraries in both single- and paired-end configuration to the community. Given the large research initiatives profiling transcriptomes of single cells in mouse [3, 17] and Human, such as the Human Cell Atlas [18], achieving high-quality and cost-effective methods is paramount. Our standardized resource can be utilized for investigating technical biases and for benchmarking scRNA-seq protocols and computational methods.

## Additional files

Additional file 1: Supplementary figures. (PDF 1140 kb)

Additional file 2: Supplementary methods. (PDF 608 kb)

Additional file 3: **Table S1.** The sequencing throughput and costs for single- and paired-end reads across both Illumina and BGISEQ-500 platform. (XLSX 9 kb)

Additional file 4: **Table S2.** Single-cell library statistics computed from raw *paired-end* sequencing reads for mESCs using SMARTer and Smart-seq2 protocols and sequenced across HiSeq2500 and BGISEQ-500 platforms. (CSV 50 kb)

Additional file 5: **Table S3.** Single-cell library statistics computed from randomly downsampled 1 million *paired-end* sequencing reads for mESCs performed using SMARTer and Smart-seq2 protocols and sequenced across HiSeq2500 and BGISEQ-500 platforms. (CSV 48 kb)

Additional file 6: **Table S4.** Single-cell library statistics computed from raw *paired-end* sequencing reads for mESCs and K562 using plate-based Smart-seq2 protocol and sequenced across HiSeq4000 and BGISEQ-500 platforms. (CSV 87 kb)

Additional file 7: **Table S5.** Single-cell library statistics computed from randomly downsampled 1 million *paired-end* sequencing reads for mESCs and K562 using plate-based Smart-seq2 protocol and sequenced across HiSeq4000 and BGISEQ-500 platforms. (CSV 82 kb)

Additional file 8: **Table S6.** Single-cell library statistics computed from raw *single-end* sequencing reads for mESCs and K562 using plate-based Smart-seq2 protocol and sequenced across HiSeq4000 and BGISEQ-500 platforms. (CSV 75 kb)

Additional file 9: **Table S7.** Metadata for all single-cell libraries profiled in this manuscript. Each cell is labeled with a sample id (accession number), protocol, place of experiment, read type, and sequencing platform.
