# Supplementary material for: Comparative analysis of sequencing technologies for single-cell transcriptomics
Source: Genome Biol. 2019 Apr 9;20:70. doi: 10.1186/s13059-019-1676-5 (PMC6454680; doi:10.1186/s13059-019-1676-5)
Supplement: Supplementary file 1 — Supplementary figures. (PDF 1140 kb) [file 13059_2019_1676_MOESM1_ESM.pdf]

Fig. S1

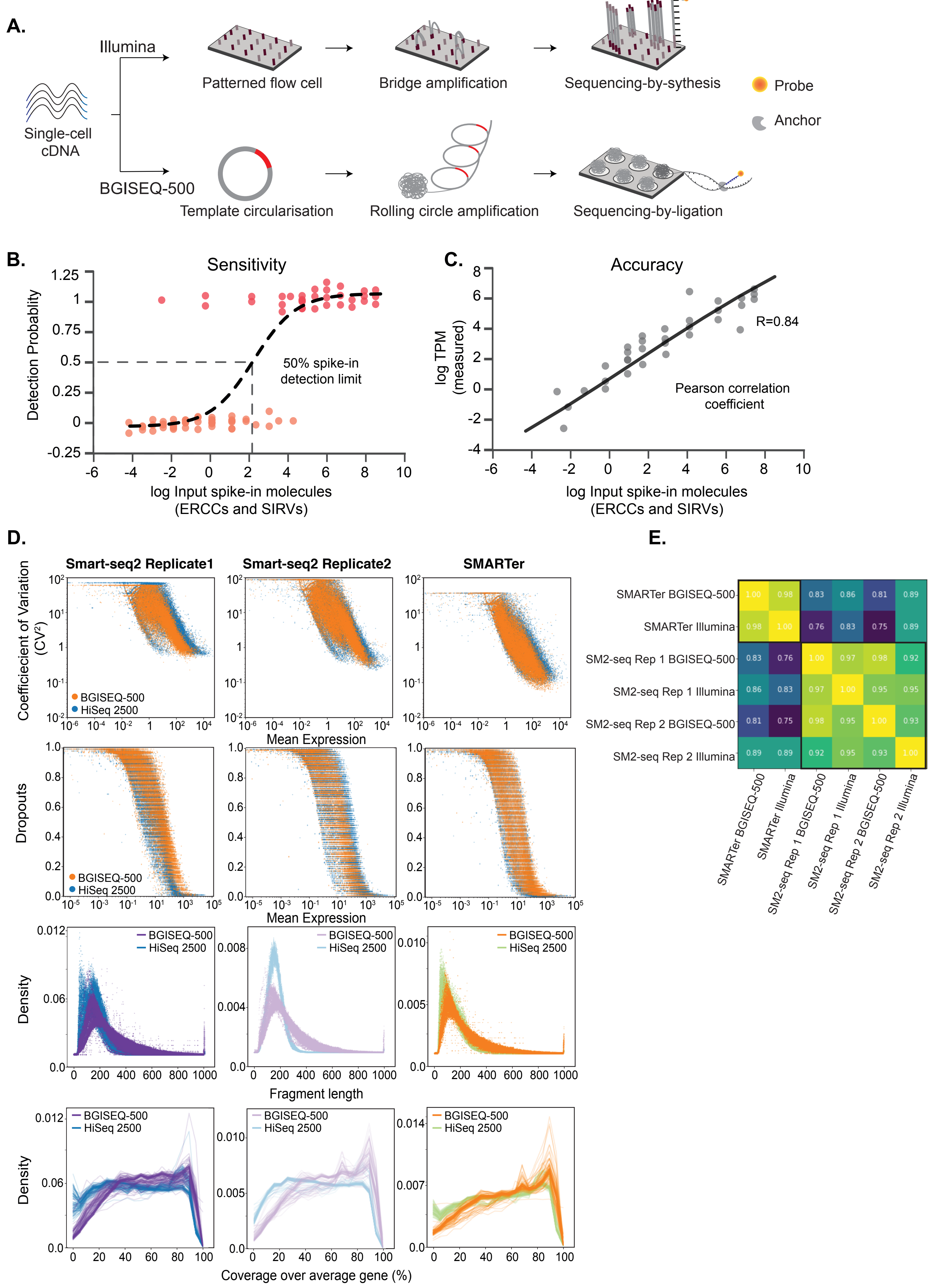
A.
Illumina
Patterned flow cell
Bridge amplification
Sequencing-by-sythesis
Probe
Anchor
Single-cell cDNA
BGISEQ-500
Template circularisation
Rolling circle amplification
Sequencing-by-ligation
B.
Sensitivity
Detection Probablity
50% spike-in detection limit
log Input spike-in molecules (ERCCs and SIRVs)
C.
Accuracy
log TPM (measured)
R=0.84
Pearson correlation coefficient
log Input spike-in molecules (ERCCs and SIRVs)
D.
Smart-seq2 Replicate1
Smart-seq2 Replicate2
SMARTer
Coefficiecient of Variation (CV²)
BGISEQ-500
HiSeq 2500
Mean Expression
Dropouts
Mean Expression
Density
Fragment length
Density
Coverage over average gene (%)
E.
SMARTer BGISEQ-500
SMARTer Illumina
SM2-seq Rep 1 BGISEQ-500
SM2-seq Rep 1 Illumina
SM2-seq Rep 2 BGISEQ-500
SM2-seq Rep 2 Illumina
1.00 0.98 0.83 0.86 0.81 0.89
0.98 1.00 0.76 0.83 0.75 0.89
0.83 0.76 1.00 0.97 0.98 0.92
0.86 0.83 0.97 1.00 0.95 0.95
0.81 0.75 0.98 0.95 1.00 0.93
0.89 0.89 0.92 0.95 0.93 1.00

Supplementary Figure 1: (A) Schematic description of library preparation and sequencing for both Illumina and BGISEQ-500 platform. Briefly in Illumina sequencing, the fragmented and barcoded template is bound to flow cell using complementary immobilized primers. The free-ends of bound fragments bind to nearby primers to form bridge structures (clusters). This process is repeated for second strand synthesis, leading to >1million template copies. The actual sequencing occurs by synthesis, where primers, DNA polymerase and modified nucleotides are added to flow cell. In each cycle, the fragments within clusters bind to a complementary modified nucleotide with cleavable fluorophore followed by flow cell imaging and cleavage of modified base. The cycles of nucleotide addition, elongation and cleavage are repeated to determine template sequence.

In BGISEQ-500, the fragmented, size-selected template undergoes sequential rounds of initial adaptor ligation, circularization and cleavage. The circularised template undergoes rolling circle amplification (RCA) to produce large DNA concatemers (DNBs) that are immobilized and sequenced on a flow cell. In cPAS-based sequencing, each DNB is bound to a complementary anchor with a fluorescent-probe to determine to identify template base. Each cycle consists of probe removal, anchor re-ligation different probe and base detection. These cycles are repeated for each initial adaptor attached to circularised template.

(B and C). Overview of performance metrics for comparing scRNA-seq protocols and sequencing platforms. **(B)** Sensitivity is described as the minimum number of input molecules (spike-in), where the detection probability is 50%. **(C)** Accuracy is calculated as the Pearson correlation between estimated expression (TPMs) and input spike-in concentrations (ground-truth). **(D)** Different gene expression metrics plotted for matched single-cells for both SMARTer and Smart-seq2 replicates from both Illumina and BGISEQ-500 platform. (**D**: row 1) Averaged gene expression variation (squared Coefficient of variation: CV^2) plotted against mean expression (log-space) across all single-cells in both platforms. (**D**: row 2) Averaged gene dropout rates across different protocols from Illumina and BGISEQ-500 platform. (**D**: row 3) Inferred fragment lengths from all paired-end reads for different protocols from both Illumina and BGISEQ-500 platform. (**D**: row 4) Estimated read coverage over averaged gene from all paired-end reads. BGISEQ-500 reads have slight bias towards 3' ends of genes, but uniform coverage across gene body. All analysed metrics between both sequencing platforms are highly similar. **(E)** Pseudobulk correlation of single-cells from different protocols and sequencing platforms. The correlations between sequencing platforms is quite high (R=0.93-0.98), with lower correlation between scRNA-seq protocols (R=0.75-0.98), due to technical protocol differences.

Fig. S2

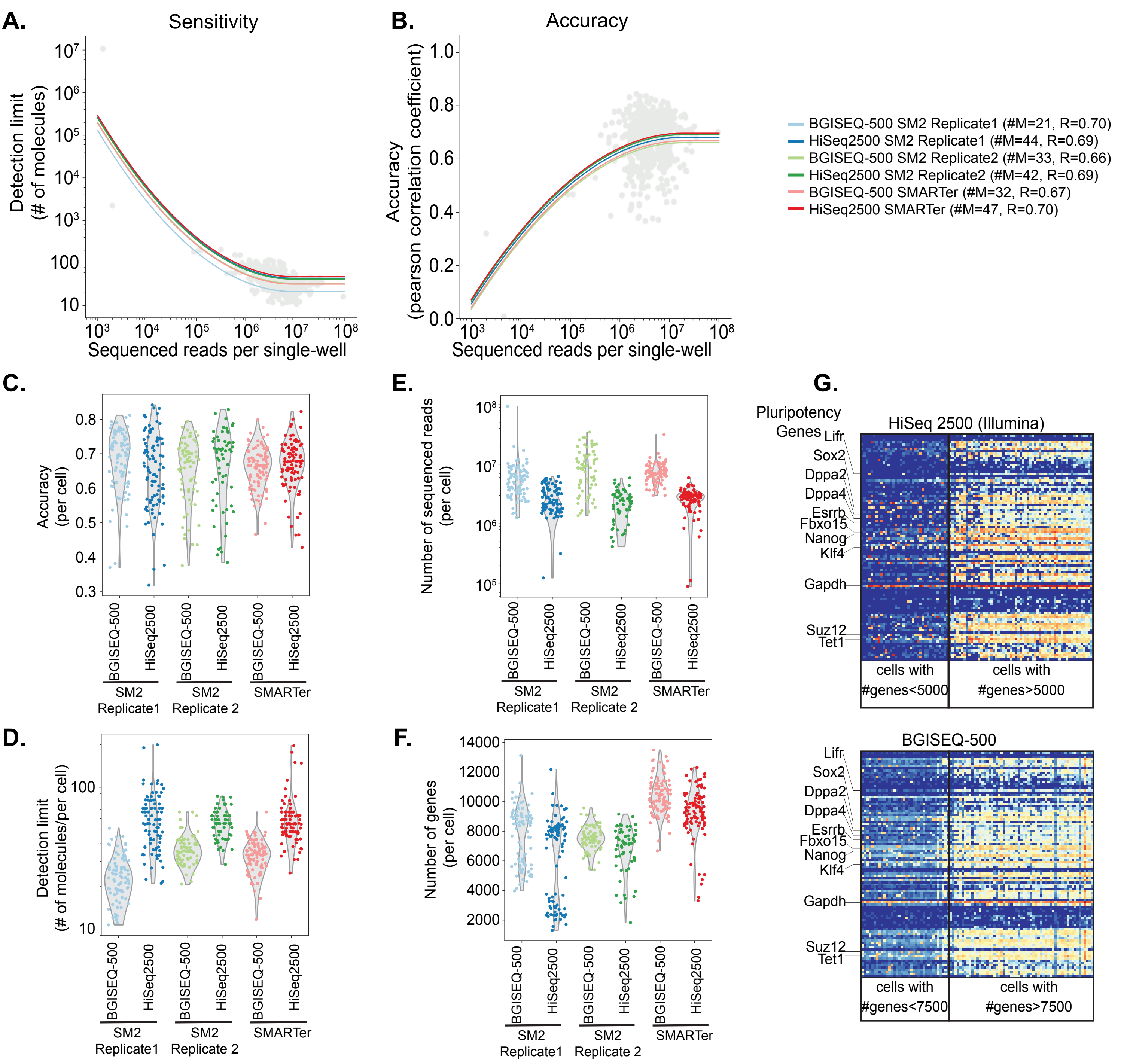
A.
Sensitivity
Detection limit (# of molecules)
Sequenced reads per single-well
B.
Accuracy
Accuracy (pearson correlation coefficient)
Sequenced reads per single-well
BGISEQ-500 SM2 Replicate1 (#M=21, R=0.70)
HiSeq2500 SM2 Replicate1 (#M=44, R=0.69)
BGISEQ-500 SM2 Replicate2 (#M=33, R=0.66)
HiSeq2500 SM2 Replicate2 (#M=42, R=0.69)
BGISEQ-500 SMARTer (#M=32, R=0.67)
HiSeq2500 SMARTer (#M=47, R=0.70)
C.
Accuracy (per cell)
D.
Detection limit (# of molecules/per cell)
E.
Number of sequenced reads (per cell)
F.
Number of genes (per cell)
BGISEQ-500
HiSeq2500
SM2 Replicate1
SM2 Replicate 2
SMARTer
G.
Pluripotency Genes
HiSeq 2500 (Illumina)
Lifr
Sox2
Dppa2
Dppa4
Esrrb
Fbxo15
Nanog
Klf4
Gapdh
Suz12
Tet1
cells with #genes<5000
cells with #genes>5000
BGISEQ-500
cells with #genes<7500
cells with #genes>7500

Supplementary Figure 2: (A-B) Single-cell accuracy **(A)** and sensitivity **(B)** of mESC cells (without downsampling) for SMARTer and two Smart-seq2 replicates (576 samples) across both sequencing platforms. **(C-F)** Violin plot representation of individual single-cell parameters computed using raw data (without downsampling) across different protocols and sequencing platforms. **(C)** Single-cell accuracies, **(D)** single-cell sensitivities, **(E)** sequencing reads per single-cell and, **(F)** genes detected per single-cell. Each dot cooresponds to a single-cell **(G)** Heatmap with expression of pluripotency and differentiation markers for Smart-seq2 replicate 1 cells across both HiSeq2500 and BGISEQ-500 cells. A cutoff of 5000 (HiSeq2500) and 7500 genes (BGISEQ-500) was selected to distinguish subpopulations, based pluripotency markers. The right subpopulation ('pluripotent') have higher number of genes expressed including pluripotency markers, while the left subpopulation ('differentiation-like') expresses fewer and more stochastically expressed genes.

Fig. S3

A. PCA using both spike-ins

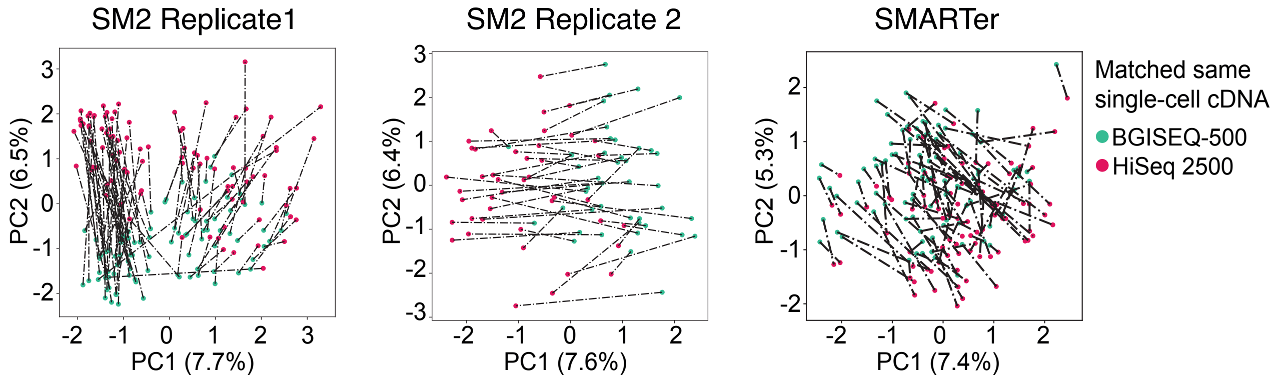

B. SM2 replicate 1 PCA

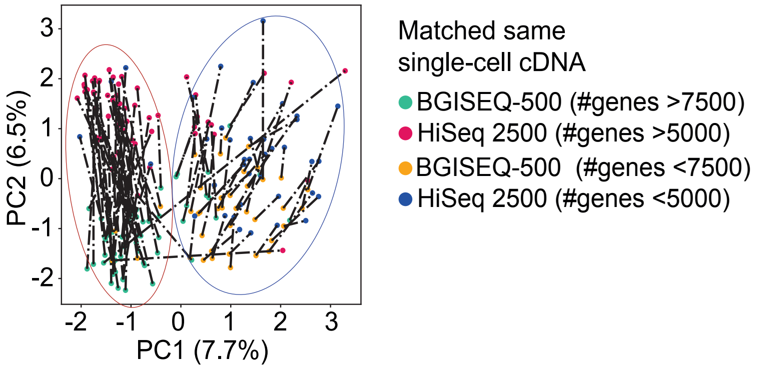

C. Single-cell sensitivity correlation using both spike-ins

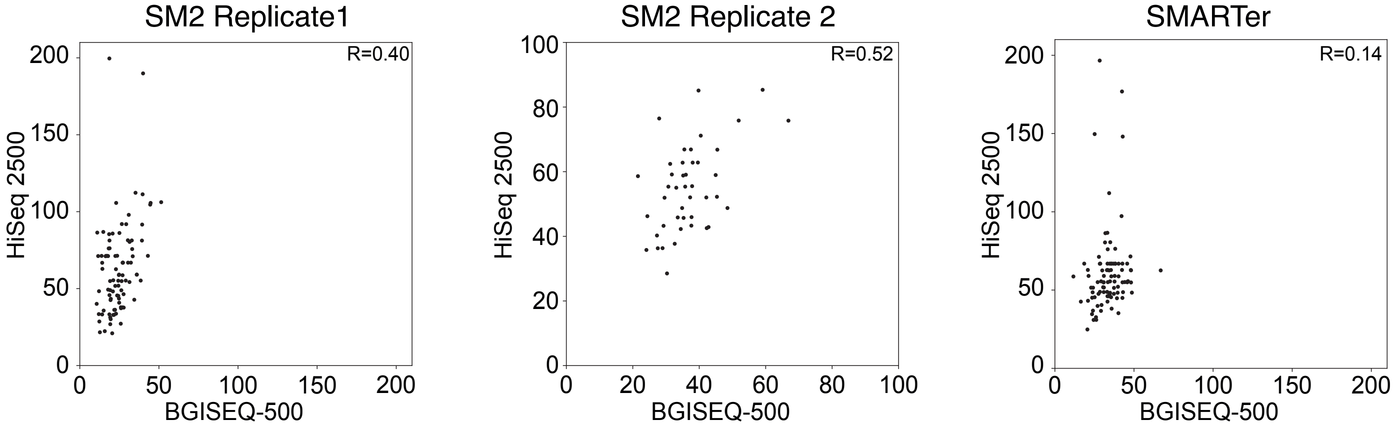

Supplementary Figure 3: (A) PCA classification of matched single-cells using only spike-in RNAs (ERCCs and SIRVs) from both sequencing platforms. Each panel represents the scRNA-seq protocol i.e. SMARTer and two replicates of Smart-seq2. The red and green colored circles indicate sequencing of matched cDNA on Illumina HiSeq2500 and BGISEQ-500 respectively. The dotted lines represent distance i.e. measure of similarity across sequencing platforms, where short distances represent highly similar expression and low variation. **(B)** PCA classification of Smart-seq2 replicate 2 re-colored by subpopulations expressing different number of genes across both platforms (as in Fig. S2G). **(C)** Single-cell correlations using only spike-in RNAs (ERCCs and SIRVs) for each scRNA-seq protocol and between sequencing platforms. The correlations (R=0.14~0.52) are quite poor owing to critical dependence of sequencing depth and skewed towards BGISEQ-500.

Fig. S4

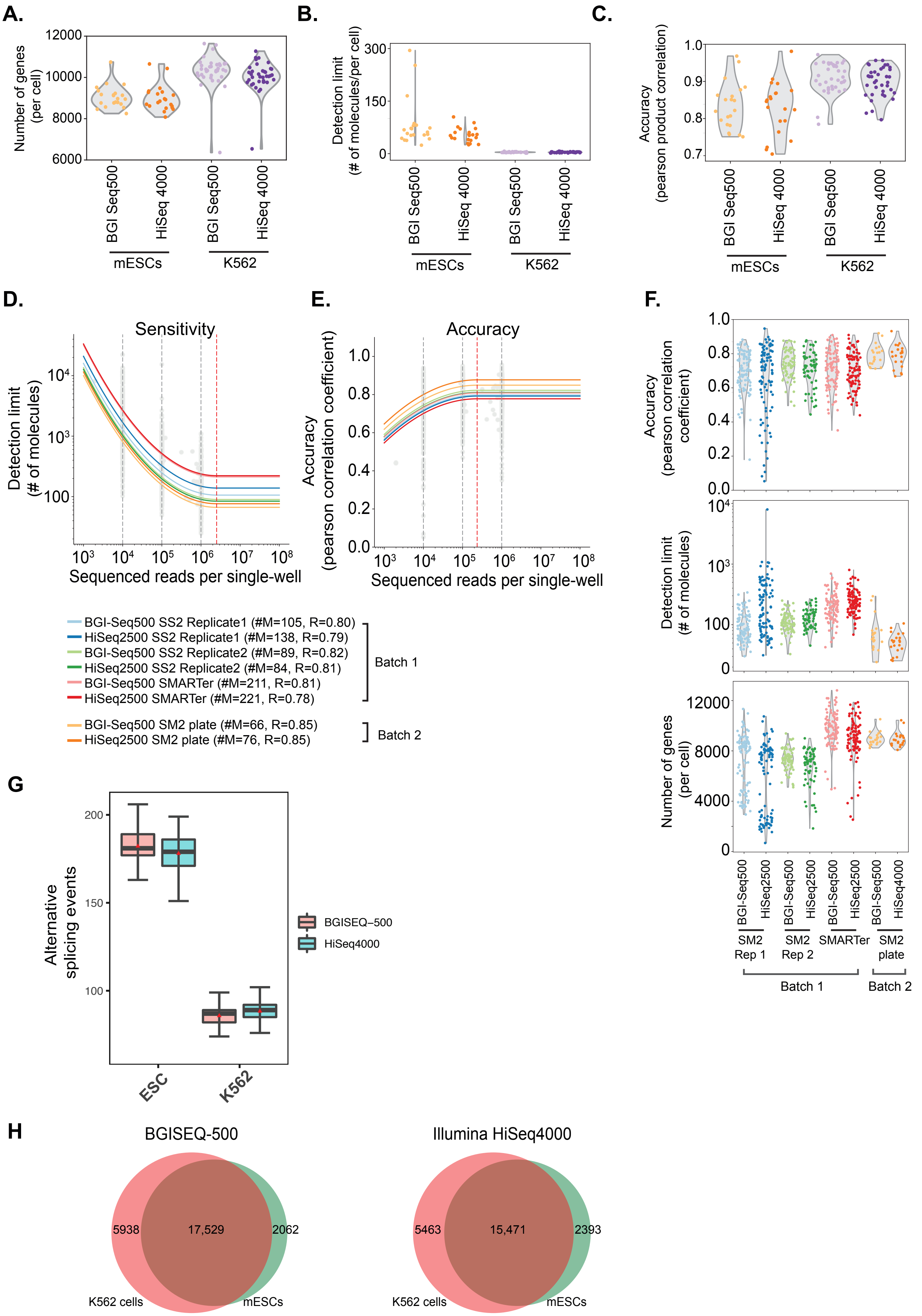
A.
Number of genes (per cell)
12000
10000
8000
6000
BGI Seq500
HiSeq 4000
BGI Seq500
HiSeq 4000
mESCs
K562
B.
Detection limit (# of molecules/per cell)
300
150
0
BGI Seq500
HiSeq 4000
BGI Seq500
HiSeq 4000
mESCs
K562
C.
Accuracy (pearson product correlation)
1.0
0.9
0.8
0.7
BGI Seq500
HiSeq 4000
BGI Seq500
HiSeq 4000
mESCs
K562
D.
Sensitivity
Detection limit (# of molecules)
Sequenced reads per single-well
E.
Accuracy
Accuracy (pearson correlation coefficient)
Sequenced reads per single-well
BGI-Seq500 SS2 Replicate1 (#M=105, R=0.80)
HiSeq2500 SS2 Replicate1 (#M=138, R=0.79)
BGI-Seq500 SS2 Replicate2 (#M=89, R=0.82)
HiSeq2500 SS2 Replicate2 (#M=84, R=0.81)
BGI-Seq500 SMARTer (#M=211, R=0.81)
HiSeq2500 SMARTer (#M=221, R=0.78)
Batch 1
BGI-Seq500 SM2 plate (#M=66, R=0.85)
HiSeq2500 SM2 plate (#M=76, R=0.85)
Batch 2
F.
Accuracy (pearson correlation coefficient)
Detection limit (# of molecules)
Number of genes (per cell)
BGI-Seq500
HiSeq2500
BGI-Seq500
HiSeq2500
BGI-Seq500
HiSeq2500
BGI-Seq500
HiSeq4000
SM2 Rep 1
SM2 Rep 2
SMARTer
SM2 plate
Batch 1
Batch 2
G
Alternative splicing events
200
150
100
BGISEQ–500
HiSeq4000
ESC
K562
H
BGISEQ-500
5938
17,529
2062
K562 cells
mESCs
Illumina HiSeq4000
5463
15,471
2393
K562 cells
mESCs

Supplementary Figure 4: (A-C) Violin plot representation of genes detected **(A)**, sensitivity **(B)** and accuracy **(C)** for single-mESCs and K562s across both sequencing platforms. Higher sequencing depth of The K562s are more deeply sequenced accounting for higher gene detection and increased sensitivity. **(D-F)** Comparison of all mESC data from different scRNA-seq protocols and technology (batch 1 and 2) from this study. The single-cell sensitivity **(D)** and accuracy **(E)** between protocols and sequencing platforms. The grey dotted lines indicate downsampling (10^4, 10^5, 10^6 reads), while red line indicates saturation per cell. **(F)** Violin plot representation of individual mESCs accuracy, detection limit and genes detected. **(G)** Box plots summarizing alternative splicing events across mESCs and K562s using downsampled 100bp paired-end reads from both sequencing platforms. **(H)** Venn diagram with genes detected (TPM>10) across both mESCs and K562s from both sequencing platforms. Most genes are commonly detected, with few mESCs and K562 specific genes.
