# Supplementary material for: Comparative analysis of sequencing technologies for single-cell transcriptomics
Source: Genome Biol. 2019 Apr 9;20:70. doi: 10.1186/s13059-019-1676-5 (PMC6454680; doi:10.1186/s13059-019-1676-5)
Supplement: Supplementary file 2 — Supplementary methods. (PDF 608 kb) [file 13059_2019_1676_MOESM2_ESM.pdf]

Supplementary Methods

Cell culture

Mouse embryonic stem cells: Wild-type E14 mouse embryonic stem cells (mESCs) were plated on 0.1% gelatin coated surfaces and cultured using Knockout DMEM (KO-DMEM, #10829, Gibco), 15% batch-tested Fetal Calf Serum (FCS, #FB-1001/500, Labtech), 1x Penicillin-Streptomycin-Glutamine (PSG, #10378-016, Gibco), 1x MEM (NEAA, #11140-035, Gibco), 2-mercaptoethanol (2-ME, #31350-010, Gibco) and 1000U Leukemia Inhibitory Factor (LIF, #ESG1107, Milipore) [1]. The mESC line (E14, #CRL-1821, ATCC) was used for BGISEQ-500 vs HiSeq4000 experiments. The mESCs were passaged every 2-3 days with daily media change.

Human K562 cells: Human K562 cells were cultured in DMEM medium supplemented with 10 % FBS and 1x Penicillin-Streptomycin-Glutamine (PSG).

Single-cell RNA-sequencing

SMARTer and Smart-seq2 on Fluidigm C1: Single-cell mESC suspension was obtained by after trypsination and passing through 30μm filter (#04-0042-2316, CellTrics). SMARTer and Smart-seq2 scripts were downloaded from Fluidigm Script Hub and run across three small C1 OpenApp IFCs (5-10μm; #100- 5759) [1]. We performed two biological replicates of Smart-seq2 across two different C1 IFCs and 1 SMARTer run, processed them independently. Two different RNA spike-ins were used (i) 92 ERCC spike-ins (#4456740, Lot# 1411014, Ambion) and (ii) 69 SIRV spike-ins (E2 SIRV, #SKU025.03, Lexogen) were mixed together (0.5μl 1:500 diluted ERCCs + 0.6μl 1:500 diluted SIRVs). We used 9μl from the total lysis buffer SMARTer (20μl) and Smart-Seq2 (27μl) to each OpenApp C1 IFCs. The subsequent steps (cell lysis, cDNA synthesis by reverse transcription and PCR reaction) are performed as described on Fluidigm ScriptHub.

Plate-based Smart-seq2: The Plate based Smart-seq2 was performed as described [2]. Briefly, single mESCs and human K562 cells were sorted into individual wells of 96-well plate containing with 4μl lysis buffer (10μM oligo-dT_{30}VN, 10mM dNTP, 4 units RNase inhibitor, ERCC spike-ins (1:5,000,000 per well)). The cell lysis was performed at 72 °C for 3 minutes, Reverse transcription using superscript II (10μl final volume) and Pre-amplification using KAPA HiFi Polymerase using IS primer (25μl final volume) as described.

Single-cell cDNA quantification: The single-cell cDNA from each protocol and platform was quantified using either DNA sensitivity 12000 chips or High Sensitivity DNA chips for 2100 Bioanalyzer (Agilent). The plate-based samples were purified using 0.9x AMPure XP magnetic beads (Beckman Coulter).

Library Preparation

Illumina HiSeq 2500: Single cell cDNA (0.3 ng/μl) from each of the 3-C1 IFCs (SMARTer and two Smart-Seq2 replicates) were independently tagmented and pooled separately using the 96 dual barcoded indices (Illumina; FC-131-1002) and the Illumina Nextera XT DNA sample preparation kit (Illumina; FC-131-1096). The resulting 3 libraries (SMARTer and two Smart-

Seq2 replicates) were cleaned and purified using 0.9x AMPure XP magnetic beads as described in Fluidigm protocol (100-5950) and Smart-seq2 protocol, followed by quantification of final library. Each library was sequenced across 1 lane of HiSeq V4 (Illumina) using 75bp/125bp paired-end sequencing at Wellcome Sanger Institute, Cambridge, UK.

Illumina HiSeq 4000: Single cell cDNA (1 ng/µl) from each plate-based Smart-seq2 runs of mESCs and K562 cells were independently tagmented at 55 °C for 7 minutes using in-house Tn5 transposase in a 20µl reaction volume/well. Tagmentation was stopped by adding 5µl of 0.1% SDS per well and 5-minute incubation at room temperature. PCR was performed using TruePrep Library kit (Vazyme) containing Amplify Enzyme, Primer 1-2, N5 and N7, for Illumina sequencing. The resulting 2 libraries (mESCs and K562s) were cleaned and purified using 1x AMPure XP magnetic beads, followed by quantification of final library. Each library was sequenced across 1 lane of HiSeq V4 (Illumina) using 100bp paired-end sequencing at BGI, China.

BGISEQ-500: Single cell cDNA (1 ng/µl) from each Fluidigm based C1 IFCs (mESCs: SMARTer and 2 Smart-seq2 replicates) and plate-based Smart-seq2 (mESCs and K562s) were independently tagmented using in-house Tn5, followed by PCR amplification using using KAPA HIFI polymerase and primer set (PhoAd153 *forward* and *reverse* primer and PhoAd153 *forward-tag* and *reverse-tag)*. The PCR steps were: Denaturation (95°C for 3 minutes), 15 cycles of Denaturation (98°C for 20 seconds), annealing (60°C for 15 seconds) and elongation (72°C for 25 seconds), and final elongation (72°C for 5 minutes). The libraries were cleaned, purified and size selected using 0.8x and 0.2x AMPure XP magnetic beads, and eluted in 25µl sterile nuclease-free water. This initial tagmentation in BGISEQ-500 protocol is a part of single-cell barcoding and not actual library preparation. For sequencing libraries, the single-cell barcoded cDNA libraries were denatured in presence of splint oligo, and circularized by incubating with T4 DNA ligase (252 Units) at 37 °C four 1 hour in a 120µl final volume. Linear DNA was removed by exonuclease digestion. The libraries were then incubated with PEG32 at room temperature for 10 minutes, followed by final elution in 40µl nuclease free water and sequencing on BGISEQ-500. All the single-end (50bp and 100bp reads) and paired-end (100bp) scRNA-seq data sequenced across BGISEQ-500 are included in Table S3 and Table S4.

scRNA-seq data processing

Data processing: The raw sequencing data and quality scores for the HiSeq 2500 were assessed based on Wellcome Sanger Institute sequencing quality control guidelines. For the HiSeq4000 and BGISEQ-500, the read quality was assessed using FastQC, overrepresented sequences and adapters (>0.5% of total reads) were filtered using Cutadapt [3].

For each demultiplexed scRNA-seq run from different sequencing runs, we quantified relative abundances using Salmon 0.8.2 [4], with library type parameter -l A and the optional flag '*--posBias --gcBias --writeUnmappedNames --numBiasSamples 100000 --numAuxModelSamples 100000 --numPreAuxModelSamples 100000*'. The Salmon transcriptome indices were built by adding 'ERCC and SIRV' sequences to cDNA sequences from Ensembl 90 cDNA annotation of GRCm38 [5].

For the mESC data (Figure 1), we generated 576 matched libraries from 288 single-cells for BGISEQ-500 and HiSeq2500 sequencing respectfully. Aftering filtering and quality control of libraries, the remaining 256 libraries (HiSeq 2500) and 263 libraries (BGISEQ-500) were used for analysis.

**Cell type**	**Unique cells**	**Unique libraries**	**BGISEQ-500**	**Illumina HiSeq4000**
			PE100	*PE100*
**mESC**	288	576	288	288

For the mESC and K562 data (Figure 2), we generated 721 libraries from 180 single-cells for BGISEQ-500 and HiSeq2500 sequencing respectfully.

**Cell type**	**Unique cells**	**Unique libraries**	**BGISEQ-500**			**Illumina HiSeq4000**
			SE50	*SE100*	*PE100*	*PE100*
**K562**	98	395	126	123	81	65
**mESC**	82	326	108	84	78	56

Performance metrics (Accuracy and Sensitivity): Similar to our previous framework [1], each spike-in with estimated TPM>1 are considered as detected. The *accuracy* is determined using Pearson product correlation between input spike-in concentration and measured TPM expression. The input concentration is known *a priori*, and is calculated multiplying the molecules per microlitre (from manufacturer) with the volume used and the final dilution in lysis mix of scRNA-seq protocol. The accuracy is only calculated, when > 7 spike-ins types are identified. The *sensitivity* is calculated using a logistic regression model (below) and as previously described [1]. The spike-in detecting probability at a given input level is modelled as:

$$p\ (detected) = \frac{1}{1 + e^{-(a*\log(M_i) + b)}} + \epsilon$$

We used Python *scikit-learn* package with class *LogisticRegression* for logistic regression and *liblinear solver* (fit_intercept = True) for fitting.

The detection limit was chosen as the molecular abundance where the logistic regression model passes 50% detection probability.

$$detection\ limit\ = -\frac{b}{a}$$

To model the relationship between sequencing depth and performance metrics (*sensitivity and accuracy*) for individual protocols, we use a linear model with a quadratic read depth term to capture diminishing returns on investment [1]. The effect of read-depth is considered global with a performance parameter for each protocol.

$$metric_i = a^2 * \log(reads_i) + b * \log(reads_i) + performance_{protocol} + \epsilon$$

Both accuracy and sensitivity saturate when

$$\log(reads) = -\frac{b}{2a}$$

The linear models were fitted using OLS regression function in Python package *statsmodels*.

Downsampling: The raw reads from each single-cell were randomly downsampled to 10^6, 10^5 and 10^4 reads. We used the downsampled reads to assess the sensitivity and accuracy of spike-ins within each cell.

Principal Component Analysis: The PCA was performed using *sklearn* function within *scikit-learn* package, after scaling the expression matrix. The correlation values were calculated with the *linregress* function in *scipy.stats* package.

Coverage and fragment distribution: The coverage rate and fragment distribution were extracted from the Salmon output. We use auxiliary file '*obs3_pos.gz*' using the results smoothed by the medium window size. We also calculated gene body coverage from STAR aligned results using RSeQC (geneBody_coverage.py) after sorting and indexing the bam files with samtools. The reference housekeeping genes used in the calculation was downloaded from the RSeQC website (https://sourceforge.net/projects/rseqc/files/BED/Mouse_Mus_musculus/). The density of each cell is normalized to 1 for comparison.

Alternative Splicing Events (ASE): The splicing alterations were detected using junction reads from the output of STAR through Outrigger (v1.1.1) [6]. Reads were firstly mapped to transcriptome using STAR (2.5.3a) using arguments "*--readFilesCommand zcat --outSAMtype BAM SortedByCoordinate --sjdbOverhang 49*" [7] using mouse (GRCm38) and human (GRCh38) reference genome. The output 'SJ.out.tab' file was then pre-processed by '*Outrigger index*' and validated using '*Outrigger validate'* using splice sites features. We used '*Outrigger psi'* to score '*percent spliced-in*' (Psi/Ψ) scores for skipped exon (SE) and mutually exclusive exon (MXE) events.

Supplementary References

1. Svensson V, Natarajan KN, Ly L-H, Miragaia RJ, Labalette C, Macaulay IC, et al. Power analysis of single-cell RNA-sequencing experiments. Nat Methods. 2017;14:381–7.

2. Picelli S, Faridani OR, Björklund AK, Winberg G, Sagasser S, Sandberg R. Full-length RNA-seq from single cells using Smart-seq2. Nat Protoc. 2014;9:171–81.

3. Martin M. Cutadapt removes adapter sequences from high-throughput sequencing reads. EMBnet.journal. 2011;17:10.

4. Patro R, Duggal G, Love MI, Irizarry RA, Kingsford C. Salmon provides fast and bias-aware quantification of transcript expression. Nat Methods. 2017;14:417–9.

5. Zerbino DR, Achuthan P, Akanni W, Amode MR, Barrell D, Bhai J, et al. Ensembl 2018. Nucleic Acids Res. 2018;46:D754–61.

6. Song Y, Botvinnik OB, Lovci MT, Kakaradov B, Liu P, Xu JL, et al. Single-Cell Alternative Splicing Analysis with Expedition Reveals Splicing Dynamics during Neuron Differentiation. Mol Cell. 2017;67:148–61.e5.

7. Dobin A, Gingeras TR. Optimizing RNA-Seq Mapping with STAR. Methods Mol Biol. 2016;1415:245–62.
